# LCVP, The Leipzig catalogue of vascular plants, a new taxonomic reference list for all known vascular plants

**DOI:** 10.1038/s41597-020-00702-z

**Published:** 2020-11-26

**Authors:** Martin Freiberg, Marten Winter, Alessandro Gentile, Alexander Zizka, Alexandra Nora Muellner-Riehl, Alexandra Weigelt, Christian Wirth

**Affiliations:** 1 Systematic Botany and Functional Biodiversity / Botanical Garden, Institute of Biology, Leipzig University, Johannisallee 21-23, 04103 Leipzig, Germany; 2 German Centre for Integrative Biodiversity Research (iDiv) Halle-Jena-Leipzig, sDiv – Synthesis Centre, Deutscher Platz 5e, Leipzig, 04103 Germany; 3 Naturalis Biodiversity Center, Darwinweg 2, 2333 CR Leiden, The Netherlands; 4 Molecular Evolution and Plant Systematics & Herbarium (LZ), Institute of Biology, Leipzig University, Johannisallee 21-23, 04103 Leipzig, Germany; 5 Max Planck Institute for Biogeochemistry, PO Box 100164, 07701 Jena, Germany

**Keywords:** Plant evolution, Plant sciences

## Abstract

The lack of comprehensive and standardized taxonomic reference information is an impediment for robust plant research, e.g. in systematics, biogeography or macroecology. Here we provide an updated and much improved reference list of 1,315,562 scientific names for all described vascular plant species globally. The Leipzig Catalogue of Vascular Plants (LCVP; version 1.0.3) contains 351,180 accepted species names (plus 6,160 natural hybrids), within 13,460 genera, 564 families and 84 orders. The LCVP a) contains more information on the taxonomic status of global plant names than any other similar resource, and b) significantly improves the reliability of our knowledge by e.g. resolving the taxonomic status of ~181,000 names compared to The Plant List, the up to date most commonly used plant name resource. We used ~4,500 publications, existing relevant databases and available studies on molecular phylogenetics to construct a robust reference backbone. For easy access and integration into automated data processing pipelines, we provide an ‘R’-package (*lcvplants*) with the LCVP.

## Abstract (summary)

**Table:** 

Measurement(s)	Plant Taxonomy
Technology Type(s)	digital curation
Sample Characteristic - Organism	Tracheophyta

Machine-accessible metadata file describing the reported data: 10.6084/m9.figshare.13013651

## Background & Summary

Due to substantial progress in the last decade in improving plant taxonomy with phylogenetic findings, an updated global taxonomic reference list was urgently required. To date, the most commonly used reference list of vascular plant names is *The Plant List* (TPL, http://www.theplantlist.org/), hosted by the Royal Botanic Gardens, Kew. TPL contains 1,166,054 vascular plant names, including 308,397 accepted names, 304,419 of them angiosperms. ~760,000 names of TPL are synonyms, including 244,017 unresolved names. The here presented Leipzig Catalogue of Vascular Plants (LCVP) updates significantly the global knowledge of plant names not only compared to TPL (see Table 1) and thus is a major improvement for global plant research. It is based on existing databases (see Online-only Table 1) and an additional 4,500 publications (see the full literature package consisting of three different files as part of the publicly available LCVP data set at https://idata.idiv.de/ddm/Data/ShowData/1806 and Step 2 below for more details), which helped to clarify the status of plant names (i.e. accepted, synonym, taxonomic placement; see Methods). In the end, 4,059 publications provided relevant and robust additional information, e.g. changes in names and/or their status. A guiding principle during the compilation of the LCVP was to avoid polyphyletic genera, which are frequent in TPL, either by splitting genera (e.g. separating *Goeppertia* from *Calathea*) or fusing them (e.g. *Stapelia* and *Duvalia* in *Ceropegia*). However, we did not recombine any species name in the LCVP and in cases of unclear phylogenetic position of genera, we used the conservative (i.e. existing) name.

**Table 1:** Summary of information content in the LCVP and name differences between LCVP and TPL.

	TPL	LCVP
Plant names	**1,166,054**	**1,315,562**
Accepted species names (excl. infraspecific taxa)	**308,397**	**351,180**
Synonyms	**760,744**	**846,279**
Accepted infraspecific names	20,719	48,257
Accepted Genera	12,660	13,460
Accepted Families	473	564
All Genera	22,830	32,986
Unresolved names	244,017	63,072
**TPL - LCVP comparison**	**Number of records**	
Identical names	841,151	
Additional names in LCVP not being in TPL	149,462	
Families in LCVP not being in TPL	96	
Families in TPL not being in LCVP	8	
Genera in LCVP not being in TPL	2,764	
Genera in TPL not being in LCVP	1,954	
Resolved in LCVP - unresolved in TPL	172,354	
Accepted in LCVP - Synonym in TPL	60,321	
Synonym in LCVP - Accepted in TPL	177,570	
Different authors in LCVP	27,824	
Different synonym in LCVP	64,703	
Different orthography in LCVP	4,962	

Taxonomists, ecologists and conservation biologists often work with many species (names) and cannot keep pace with the rapid progress in (plant) systematics, boosted by molecular phylogenetic methods^1^. These researchers often rely on taxonomic reference lists as tools to translate taxa names to accepted species names via accepted synonyms.

Comprehensive taxonomic lists, such as the LCVP^2^, are essential to standardize names in databases compiled from various sources, relying on a robust ‘translation’ of species names into one scheme. The TRY database of functional plant traits (TRY^3^; www.try-db.org) is one of the most prominent examples containing trait information for about 150,000 vascular plant species. Other global databases using plant name reference lists focus on plant co-occurrence patterns, such as sPlot containing about 1,1 million vegetation surveys (^4^~55,000 species), or use any plant species occurrence information, such as the Global Biodiversity Information Facility (~315,000 vascular plant species; www.gbif.org), of the Botanical Information and Ecology Network (BIEN^5^: ~348,000). The Global Inventory of Floras and Traits (GIFT^6^: ~268,000; http://gift.uni-goettingen.de/home) or the inventory of the Global Naturalized Alien Flora (GloNAF^7^~14,000; glonaf.org) focus on plant distribution information from regional floras or floristic inventories.

Generally, such databases were compiled from heterogeneous data sources varying in time of publication and place of origin. The underlying sources may be primary or secondary literature - using work of scientists with excellent to no plant taxonomic background, thus combining data with various degrees of complexity and uncertainty. The merging of these databases works via species identities and thus depends on the use of accepted species names. These databases typically tap phylogenetic information contained in taxonomic references lists via available tools supporting automated matching and error checking (i.e. taxon scrubbing). There is a variety of R packages (e.g. taxonstand^8^; taxize^9^; RBIEN^10^) or online tools (e.g. Global Name Resolver http://resolver.globalnames.org/ or the Taxonomic Name Resolution Service^11^ http://tnrs.iplantcollaborative.org/TNRSapp.html) supporting researchers to check their taxonomic information (see^12^ for a review on some of those tools). However, most of these tools rely on TPL as a reference list, which has not been updated for almost a decade and originated in a time when phylogenetic information on many genera did not exist.

Global taxonomic name databases are useful in their own right, and jointly create synergies that have transformed ecology into a synthetic and global science, and can help identifying knowledge gaps^13^. For example, functional biogeography combines information on community composition, plant species distribution and functional traits of the component species to make inferences on determinants of global trait distribution^14^. While there is high potential for exciting research using up-to-date taxonomic information, it can be only as good as the input data and the ability of the user to understand the advantage and shortcoming of the data coming from those resources. For example, missing taxonomic background often leads to neglecting the importance of citing authors of names and inevitably leads to inconsistencies when data from different sources are matched. LCVP^2^ shows that when matching plant taxonomic names without author names, results could have up to 10% mismatches (i.e. ~10% of all LCVP plant taxa names are identical but ultimately refer to different accepted plant taxa).

## Methods

The creation of the LCVP involved three major steps. (1) We did a thorough search of available and relevant plant taxonomic databases (Online-only Table 1) to collate a raw data table of existing plant names (see Step 1: Producing the raw data table). This table included many contradictory opinions in taxonomic placement of species. (2) Based on additional information in ~4,500 publications and the reliability, timeliness and quality of relevant scientific evidence in this literature we, decided for each name, whether that name is in LCVP accepted, synonymous or unresolved (see for more details Step 2: Decision making). Additionally, we harmonized and corrected taxonomic names orthographically. (3) We implemented the LCVP in an R package (LCVP) which is accessible under a MIT license from GitHub (https://github.com/idiv-biodiversity/LCVP) and will ensure a coherent versioning of the list and future updates. Furthermore, we provide a utility function to use LCVP for taxonomic name resolution (lcvplants), which is also available under the same license from GitHub (https://github.com/idiv-biodiversity/lcvplants).

### Step 1: Producing the raw data table

TPL provided the core of the raw data table for published vascular plant names, primarily supplemented by the International Plant Names Index (IPNI, https://www.ipni.org/). IPNI provides a list of published names and their source, but does not provide any information on accepted or synonymous names. We used additional major and minor databases (see Online-only Table 1 and http://www.ville-ge.ch/musinfo/bd/cjb/africa/recherche.php?; http://gentian.rutgers.edu/classNEW123.htm; http://botany.si.edu/gesneriaceae/checklist/result.cfm; http://www.systax.org/; https://parasiticplants.siu.edu/ListParasites.htm; https://floramalesiana.org/new/; http://www.plantsoftheworldonline.org/; www.catalogueoflife.org/annual-checklist/2019; http://www.omnisterra.com/bot/cp_home.cgi; https://rbg-web2.rbge.org.uk/diptero/diptax.html; https://compositae.landcareresearch.co.nz/; http://www.cvh.ac.cn/cvh6/view/index.php; http://cichorieae.e-taxonomy.net/portal/; http://ww2.bgbm.org/EuroPlusMed/query.asp; http://floradobrasil.jbrj.gov.br/reflora/listaBrasil/ConsultaPublicaUC/ConsultaPublicaUC.do#CondicaoTaxonCP; http://www.melastomataceae.net/MELnames/; https://plants.usda.gov/java/; https://collections.nmnh.si.edu/search/botany/; http://posa.sanbi.org/sanbi/Explore; http://palmweb.org/) which we have chosen based on their availability, on our expert judgement on comprehensiveness, and whether they contained information if taxa names are accepted or not (see Online-only Table 1 for a table of used databases). All additional names and potential synonyms found in those databases were incorporated in the raw data table.

### Step 2: Decision making

The raw data table with more than two million entries of plant taxa names contained a high number of orthographic errors, inconsistencies and contradictory opinions concerning the status of the names. A rough guideline for the acceptance of names was a subjective assignment of quality and reliability to the source. Generally, changes were only applied when the authors of the respective publications were clearly suggesting those changes. We ascribed a higher reliability rank (e.g. for conflicting information) usually to the most recent publications. Additionally, when conflicting information appeared we usually used information from publications with a) a more thorough literature section and b) a more comprehensive synonymy history than to those without. A complete synonymy history should include and properly cite not only the latest accepted taxon, but also the depending taxonomic history of all names connected to this taxon (e.g. if it is a recombined taxon) with all homonymic (i.e. species epitheton is the same) and heteronymic (i.e. genus name is the same) synonyms. Since phylogenies based on morphological data alone are prone to homoplasy, only phylogenetic studies that made taxonomical decisions also based on molecular data were taken into account. We did not create new species name combinations. In case of conflicting evidence on the phylogenetic placement or species name, due to e.g. different methods to build phylogenetic trees, species names were marked “comb.ined.” following the basionym author.

The following examples illustrate how we treated name changes: The genus *Dracaena* and *Sansevieria* are closely related^15^, where *Sansevieria* seems to be clearly nested within *Dracaena*, but the differences between both genera are continuous. Lu *et al*.^15^ separated the Hawaiian species of *Dracaena* in a new genus *Chrysodracon*, but did not recombine *Sansevieria* with *Dracaena* yet. The presented argumentation and data in^15^ were thorough and comprehensive and thus we accepted the authors arguments, kept *Sansevieria* and *Dracaena* as distinct genera and separated the Hawaiian species of *Dracaena* in the new genus *Chrysodracon*. In another case Borchsenius *et al*.^16^ showed that *Calathea* in the traditional description was polyphyletic. In order to keep *Ischnosiphon* and *Monotagma* as distinct genera, being the sister clade to a smaller *Calathea* clade including the type species, the larger clade of *Calathea* was put into the then resurrected genus *Goeppertia*. The argumentation and presentation in^16^ was robustly based on a molecular phylogeny producing well supported clades. As a consequence, we accepted the recombination of the much larger clade as suggested in^16^.

We also applied changes to the spelling of species names. Generally, we recommend to check the species names prior to automated list treatments, following the guidelines given in^17^ and the rules of the current version of the International Code of Nomenclature for algae, fungi, and plants (Shenzhen Code^18^). We followed the Shenzen Code using standardized orthography of epitheta across genera and families, e.g. *warscewiczii* (neither *warscewitzii* nor *warszewiczii*). Only upper cases from ‘A’ to ‘Z’, lower cases from ‘a’ to ‘z’ and the hyphen ‘-‘ should be used in scientific names, special characters are not valid and to be avoided (Isoëtes- > Isoetes, Köberlinia - > Koeberlinia). Authors were given in their short form as provided by IPNI. For further standardization and easier use in automated workflows, we omitted spaces within author names (C. F. W. Meissn., C.F. W. Meissn., C. F.W. Meissn. C. F. W.Meissn. - > C.F.W.Meissn.; Balf. f.- > Balf.f.). We linked names published by two authors with the ‘&’ sign (e.g. *Primula minor* Balf.f. & Kingdon-Ward). Names published by three and more authors were restricted to the first authors followed by ‘& al.’ (e.g. *Limonium irtaense* P.P.Ferrer, A.Navarro, P.Pérez, R.Roselló, Rosselló, M.Rosato & E.Laguna - > *Limonium irtaense* P.P.Ferrer & al.). This refers to the recommendation of the Shenzhen Code, Art. 46 c. We tried to include only natural hybrids (i.e. no cultivars; based on expert judgement of LCVP authors) in the LCVP. Since hybrids were not the focus of the LCVP, we only marked them with ‘_x’, either following the genus name or the epitheton to recognize them as such, but we did not give any parent taxa information.

In most cases, we adopted the names used by the taxonomic expert (i.e. reference author who is usually a person with a publication record within a certain taxonomic group). However, there are many taxa belonging to genera or species which have not been phylogenetically analyzed yet. For those, we adapted the most frequently used taxon name from the recent literature. Despite a major effort, there are still names, which we could not resolve.

As part of the LCVP data package we also provide at https://idata.idiv.de/ddm/Data/ShowData/1806 three different files related to the used literature that we used to decide upon species names to create LCVP. We provide a complete bibliography (as.bib file and as full text pdf) of all ~4,500 literature references ordered by plant families. We focused on literature published from 1994 onwards, when molecular phylogenies became widespread^19,20^. The third file is a table directly matching >104,000 individual taxa and literature, used to inform the applied name changes for the respective taxa.

### Step 3: Implementation in R

Besides providing LCVP as downloadable text table^2^ with this article, we also provide LCVP as R package for easy integration with analyses pipelines. Due to the large size of the data we provide a pure data package, LCVP, and a separate tool package, lcvplants, with a fuzzy matching algorithm for taxonomic name resolution. Both can be downloaded and installed via github. The LCVP data package solely contains three files: the dataset of plant names and their taxonomic status, a package of the literature references used to compile the list (consisting of three files) and a meta data description file. The lcvplants package contains one user-level function to perform a fast fuzzy matching for taxonomic name resolution using the LCVP data^2^. This taxonomic names resolution is implemented in a user-friendly way, and can be done with few lines of code (see https://idiv-biodiversity.github.io/lcvplants/articles/taxonomic_resolution_using_lcplants.html for a tutorial):

```

# install LCVP and lcvplants from GitHub

install.packages(“devtools”)

library(devtools)

devtools::install_github(“idiv-biodiversity/LCVP”)

devtools::install_github(“idiv-biodiversity/lcvplants”)

# load the package

library(lcvplants)

# run analyses

LCVP(“Hibiscus vitifolius”)

“‘

#### Input data

For taxonomic name resolution an individual name or a vector of names can be provided. There are no limits on the number of names submitted at a time, but we recommend to submit less than 5000 names at a time to ensure a reasonable computation time. For the input data, following the International Code of Nomenclature for algae, fungi, and plants (Shenzhen Code: https://www.iapt-taxon.org/nomen/main.php), genus, epithet, infraspecies rank, infraspecies name and authorities need to be separated by spaces (e.g. *Draba mollissima* var. *kusnezowii* N.Busch). Special characters (such as ü, á, ø, etc.) are only allowed for the authority names. Infraspecific names have to be preceded by their rank (e.g. “subsp.”, “var.”, “forma”, “ssp.”, “f.”, “subvar.”, “subf.”). The genus name and the epitheton need to be provieded; the infraspecific ranks and authority names are optional for better results. If the genus or the epitheton are composed of two words, they have to be separated by a hyphen (e.g. *Hibiscus rosa-sinensis* L.). Hybrid names use the characters ‘_x’ at the end of the genus and epithet name (e.g. *Spartocytisus_*x *filipes* Webb & Berthel., *Lycopodium habereri*_x House) annotations in other formats such as ‘x’ or ‘x_’ before the names are automatically changed into the required format. The commonly used special Unicode Character ‘ x ’ (U + 00D7) for indicating hybrids is not accepted (e.g. *Crassocephalum* x *picridifolium*).

#### Fuzzy matching

The lcvplants package performs a string comparison between the user-submitted names and LCVP using a fuzzy matching algorithm to solve orthographic errors. The fuzzy matching algorithm can be applied to the genus name, the epitheton, the infraspecific names and the authority (see Online-only Table 2 for a description of the options for customization), and runs in the following order:

1. Submitted name standardization. The submitted name is standardized into parts using a space as delimiter: The genus level (first word) and the epitheton (second word). If there are more than three words in the submitted name and the third word is any of: “subsp.”, “var.”, “forma”, “ssp.”, “f.”, “subvar.” or “subf.” the fourth term will be recognized as the infraspecies name. Otherwise all the words after the epitheton will be recognized as authority description.
2. Genus resolution with a user-specified threshold of allowed mismatches (i.e. the number of letters that can disagree between submitted and matched name).
3. Epitheton resolution. If a match for the submitted genus name is found, a similar matching will be done to find the correct epitheton.
4. Infraspecific name and authority resolution. If genus and epitheton resolution were successful, the fuzzy matching will be applied also for infraspecific names and authority names (if supplied).
5. The results for all submitted names will be combined into the output table and the results will be returned by the function and printed to the screen.

#### Output data

The output is a data.frame of the submitted and matched taxon names with additional information on the taxonomic status. If the option ‘save’ is turned active (Save = TRUE), the output will additionally be saved in a comma-separated file (.csv) in the working directory or the path specified with the ‘out_path’ option. The following list describes the columns of the output table. If a name could not be resolved, in the LCVP the respective row in the output data.frame is empty except for the ‘Submitted_Name’ and the ‘Score’ field, which gives detail information in which parts of the name could not be matched. See Online-only Table 3 for a description of the output fields.

## Data Records

LCVP^2^ contains 1,315,562 vascular plant names with 351,180 accepted species names (405,687 including infraspecific taxa) and 846,279 synonyms (Table 1). The accepted species in LCVP belong to 13,460 genera, 564 families, and 84 orders. LCVP significantly reduced the number of unresolved plant names by ~181,000 to ~63,000 (5%) taxa compared to TPL (Table 1).

## Technical Validation

We tested whether all synonyms lead to an accepted name or another synonym. One major issue with TPL is the high amount of unresolved names. A link to another name sometimes is another synonym leading to unresolved loops. LCVP only links to accepted names, not to the taxonomic predecessor. If taxon A is synonym to taxon B and it turned out, that taxon B is synonym to taxon C, the accepted name given for taxon A is taxon C, not B. We treated invalid names as synonyms and assigned them to their appropriate accepted name.

Most of the still unresolved species names in LCVP were originally published in the 19th century. There is a high probability that the majority of them are synonyms, e.g. because of historic transfer errors from one publication to the other. An extraordinarily high amount of unresolved names can be found in Asteraceae (in *Hieracium* 5,781 out of 19,300 names are unresolved, *Senecio* 682 out of 6,684, *Cirsium* 353 out of 2,162), Rosaceae (*Rubus* 4,005 out of 10,199, *Rosa* 2,298 out of 5,965, *Prunus* 509 out of 2,072, *Potentilla* 724 out of 3,954, *Crataegus* 720 out of 2,717, *Pyrus* 373 out of 1,199), Salicaceae (*Salix* 610 out of 3,867), Araceae (*Anthurium* 582 out of 2,261), and Geraniaceae (*Pelargonium* 962 out of 1,846).

### Comparison to TPL

Due to the improved name resolution and increased name information in general in LCVP compared to TPL, any work flow including taxonomic harmonization of plant names, will very likely yield more robust and reliable results for e.g. species richness patterns and matches between different data sources. For an easier comparison between LCVP and TPL, LCVP includes information whether taxa name entries are identical, differ in the cross-reference to a synonym, differ only orthographically either by the name or the author, or whether a name is new in the LCVP and not present in TPL. This unique information makes it possible for the users of TPL to update their names according to the LCVP, because all differences are clearly stated in the column ‘status’ of the LCVP.

Kew Gardens´ research effort to standardize plant names recently focuses on their new flagship program, Plants of the World Online (POWO, http://www.plantsoftheworldonline.org/), which includes a new taxonomic reference backbone (Alan Paton from Kew Gardens, pers. comm. July 2019). Given that this is becoming the successor of TPL (see http://www.plantsoftheworldonline.org/about) we also compared the available POWO list with LCVP (POWO access date: November 2018; directly provided by Kew). With ~335,000 accepted species names and ~458,000 names of vascular plants marked as synonyms in this POWO version, LCVP contains also significantly more species name information than POWO (this comparison includes only vascular plants and excludes infraspecific taxa since LCVP covers only vascular plants and this POWO version does not include taxa below species level).

TPL and the tested POWO version cover all plants, LCVP only vascular plants. With the current information we have, LCVP contains more information about vascular plant names (e.g. more resolved names, more accepted species, more synonyms) than TPL and POWO. A user is more likely to resolve a given vascular plant name with LCVP than with the given versions of TPL and POWO. Any future updated versions of LCVP and POWO will change these numbers and might strengthen different purposes of use for each reference list, and could ideally lead to a harmonized global backbone if applicable. LCVP covers also infraspecific names which are not covered in the tested POWO version. The information in LCVP to which genus a species belongs and/or thus which accepted name should be used, is based on taxonomic, but also on most recent phylogenetic (i.e. mainly genetic) information. TPL was not updated for many years, and is mainly based on taxonomic information (i.e. not molecular phylogenies). With respect to usability of LCVP, we do see advantages compared to the POWO version we tested, which to our knowledge does not offer an R package nor any other functionality of (half)automatic name checking or any fuzzy name matching functions.

## Data Availability

The LCVP generally consists of (1) the LCVP itself, available as R data package (version 1.0.3 as of July 2020) and as tab-delimited textfile file and (2) the R-package lcvplants. The LCVP version 1.0.3 is available in both Microsoft Excel and text formats in the iDiv data portal (https://idata.idiv.de/ddm/Data/ShowData/1806; 10.25829/idiv.1806-40-3009). A developmental version of the LCVP and the lcvplants package are publicly available via GitHub (https://github.com/idiv-biodiversity/lcvplants). We will constantly update the LCVP and plan to release a new version once every second to third year. We plan to closely collaborate with plant synonymy services and tools like e.g. BIEN, GNR, R packages taxonstand and taxize, to include LCVP as reference option. Requests for integrating LCVP can be made via the projects GitHub (https://github.com/idiv-biodiversity/LCVP/issues).

## Online-only Tables

**Online-only Table 1:** Freiberg *et al*., LCVP, The Leipzig catalogue of vascular plants, a new taxonomic reference list for all known vascular plants. Major sources used in compiling the raw-data list as basis for the Leipzig Catalogue of Vascular Plants (LCVP) See also refs. ^21–33^.

Source	Reference information if available	License information if available	Copyright information if available
Carnivorous Plant Database (http://www.omnisterra.com/bot/cp_home.cgi)	Carnivorous Plant Database: http://www.omnisterra.com/bot/cp_home.cgi, International Carnivorous Plant Society (2017)		Dr. Barry Rice, Science Editor of Carnivorous Plant Newsletter
Co’s Digital Flora of the Phillippines (http://www.philippineplants.org/)	Pelser, P.B., J.F. Barcelona & D.L. Nickrent (eds.). Co’s Digital Flora of the Phillippines. 2011 onwards.		Co’s Digital Flora of the Philippines
Dipterocarpaceae Data Base (https://rbg-web2.rbge.org.uk/diptero/diptax.html)	Mark Newman, (Supervisor), Edinburgh; Dipterocarpaceae Data Base at the RBGE PANDORA database system (2015)		Copyright Royal Botanic Garden Edinburgh 1998
Gentian Research Network (http://gentian.rutgers.edu/classNEW123.htm)	At 23^rd^ March 2020 not available anymore		
Gesneriaceae Research (http://botany.si.edu/gesneriaceae/checklist/result.cfm)	Not existent anymore		
Global Compositae Checklist (https://compositae.landcareresearch.co.nz/)	Flann, C (ed) 2009 + Global Compositae Checklist. Accessed 2016–2019	Use of these data allowable with due attribution [citation as mentioned]	
India Biodiversity Portal (https://indiabiodiversity.org/)	Vattakaven T, George R, Balasubramanian D, Réjou-Méchain M, Muthusankar G, Ramesh B, Prabhakar R (2016) India Biodiversity Portal: An integrated, interactive and participatory biodiversity informatics platform. Biodiversity Data Journal 4: e10279. 10.3897/BDJ.4.e10279		
International Plant Names Index	IPNI (2010). International Plant Names Index. Published on the Internet http://www.ipni.org, The Royal Botanic Gardens, Kew, Harvard University Herbaria & Libraries and Australian National Botanic Gardens. [Retrieved 1 April 2010].		
Parasitic Plant Classification (https://parasiticplants.siu.edu/ListParasites.html)	Daniel L. Nickrent, https://parasiticplants.siu.edu/ListParasites.htm, Department of Plant Biology, Southern Illinois University (2016)		Copyright © 1997 Daniel L. Nickrent
Plants of the World online (http://www.plantsoftheworldonline.org/)	Plants of the World Online. Facilitated by the Royal Botanic Gardens, Kew. Published on the Internet; http://www.plantsoftheworldonline.org/ last time assessed 01.12.2019	CC - BY	Copyright Board of Trustees of the Royal Botanic Gardens, Kew
SysTax (http://www.systax.org/)	SysTax – ein Datenbanksystem für Systematik und Taxonomie © Uni Ulm 2015		
The African Plant Database (Botanical Garden of Geneva, http://www.ville-ge.ch/musinfo/bd/cjb/africa/recherche.php?langue = an)	© 2012 Conservatoire et Jardin botaniques & South African National Biodiversity Institute		
The BrassiBase (https://brassibase.cos.uni-heidelberg.de/)	Kiefer M, Schmickl R, German DA, Lysak M, Al-Shehbaz IA, Franzke A, Mummenhoff K, Stamatakis A, Koch MA. 2014. BrassiBase: Introdcution to a novel database on Brassicaceae evolution. Plant Cell Physiol., 55(1): e3, doi:10.1093/pcp/pct158. Koch MA, German DA, Kiefer M, Franzke A. 2018. Database taxonomics as key to modern plant biology. Trends Plant Sci. 23(1): 4–6. 10.1016/j.tplants.2017.10.005		Usually webpages may be freely distributed and copied. However, it is requested that in any subsequent use of this work, BrassiBase be given appropriate acknowledgment and cited.
The Catalogue of Life (http://www.catalogueoflife.org/)	Roskov Y., Ower G., Orrell T., Nicolson D., Bailly N., Kirk P.M., Bourgoin T., DeWalt R.E., Decock W., Nieukerken E. van, Zarucchi J., Penev L., eds. (2019). Species 2000 & ITIS Catalogue of Life, 2019 Annual Checklist. Digital resource at www.catalogueoflife.org/annual-checklist/2019. Species 2000: Naturalis, Leiden, the Netherlands. ISSN 2405–884X Hassler M. (2018). World Ferns: Checklist of Ferns and Lycophytes of the World (version Apr 2018). In: Species 2000 & ITIS Catalogue of Life, 2018 Annual Checklist (Roskov Y., Abucay L., Orrell T., Nicolson D., Bailly N., Kirk P.M., Bourgoin T., DeWalt R.E., Decock W., De Wever A., Nieukerken E. van, Zarucchi J., Penev L., eds.). Digital resource at www.catalogueoflife.org/annual-checklist/2018. Species 2000: Naturalis, Leiden, the Netherlands. ISSN 2405-884X. Calonje M., Stanberg L. & Stevenson D. (eds). (2018). The World List of Cycads, online edition (version Jun 2015). In: Species 2000 & ITIS Catalogue of Life, 2018 Annual Checklist (Roskov Y., Abucay L., Orrell T., Nicolson D., Bailly N., Kirk P.M., Bourgoin T., DeWalt R.E., Decock W., De Wever A., Nieukerken E. van, Zarucchi J., Penev L., eds.). Digital resource at www.catalogueoflife.org/annual-checklist/2018. Species 2000: Naturalis, Leiden, the Netherlands. ISSN 2405-884X. Farjon A., Gardner M. & Thomas P. (2018). Conifer Database (version Jan 2014). In: Species 2000 & ITIS Catalogue of Life, 2018 Annual Checklist (Roskov Y., Abucay L., Orrell T., Nicolson D., Bailly N., Kirk P.M., Bourgoin T., DeWalt R.E., Decock W., De Wever A., Nieukerken E. van, Zarucchi J., Penev L., eds.). Digital resource at www.catalogueoflife.org/annual-checklist/2018. Species 2000: Naturalis, Leiden, the Netherlands. ISSN 2405-884X Govaerts R. (ed). For a full list of reviewers see: http://apps.kew.org/wcsp/compilersReviewers.do (2018). WCSP: World Checklist of Selected Plant Families (version Aug 2017). In: Species 2000 & ITIS Catalogue of Life, 2018 Annual Checklist (Roskov Y., Abucay L., Orrell T., Nicolson D., Bailly N., Kirk P.M., Bourgoin T., DeWalt R.E., Decock W., De Wever A., Nieukerken E. van, Zarucchi J., Penev L., eds.). Digital resource at www.catalogueoflife.org/annual-checklist/2018. Species 2000: Naturalis, Leiden, the Netherlands. ISSN 2405-884X Hassler M. (2018). World Plants: Synonymic Checklists of the Vascular Plants of the World (version Apr 2018). In: Species 2000 & ITIS Catalogue of Life, 2018 Annual Checklist (Roskov Y., Abucay L., Orrell T., Nicolson D., Bailly N., Kirk P.M., Bourgoin T., DeWalt R.E., Decock W., De Wever A., Nieukerken E. van, Zarucchi J., Penev L., eds.). Digital resource at www.catalogueoflife.org/annual-checklist/2018. Species 2000: Naturalis, Leiden, the Netherlands. ISSN 2405-884X. Rainer H. & Chatrou L.W. (eds) (2018). AnnonBase: Annonaceae GSD (version Jan 2014). In: Species 2000 & ITIS Catalogue of Life, 2018 Annual Checklist (Roskov Y., Abucay L., Orrell T., Nicolson D., Bailly N., Kirk P.M., Bourgoin T., DeWalt R.E., Decock W., De Wever A., Nieukerken E. van, Zarucchi J., Penev L., eds.). Digital resource at www.catalogueoflife.org/annual-checklist/2018. Species 2000: Naturalis, Leiden, the Netherlands. ISSN 2405-884X. Warwick S.I., Francis A. & Al-Shehbaz I.A. (2018). Brassicaceae species checklist and database (version 2, Oct 2009). In: Species 2000 & ITIS Catalogue of Life, 2018 Annual Checklist (Roskov Y., Abucay L., Orrell T., Nicolson D., Bailly N., Kirk P.M., Bourgoin T., DeWalt R.E., Decock W., De Wever A., Nieukerken E. van, Zarucchi J., Penev L., eds.). Digital resource at www.catalogueoflife.org/annual-checklist/2018. Species 2000: Naturalis, Leiden, the Netherlands. ISSN 2405-884X. Culham A. & Yesson C. (2018). Droseraceae Database (version 0.1, Dec 2008). In: Species 2000 & ITIS Catalogue of Life, 2018 Annual Checklist (Roskov Y., Abucay L., Orrell T., Nicolson D., Bailly N., Kirk P.M., Bourgoin T., DeWalt R.E., Decock W., De Wever A., Nieukerken E. van, Zarucchi J., Penev L., eds.). Digital resource at www.catalogueoflife.org/annual-checklist/2018. Species 2000: Naturalis, Leiden, the Netherlands. ISSN 2405-884X. Roskov Y., Zarucchi J., Novoselova M. & Bisby F.(†) (eds). (2018). ILDIS World Database of Legumes (version 12, May 2014). In: Species 2000 & ITIS Catalogue of Life, 2018 Annual Checklist (Roskov Y., Abucay L., Orrell T., Nicolson D., Bailly N., Kirk P.M., Bourgoin T., DeWalt R.E., Decock W., De Wever A., Nieukerken E. van, Zarucchi J., Penev L., eds.). Digital resource at www.catalogueoflife.org/annual-checklist/2018. Species 2000: Naturalis, Leiden, the Netherlands. ISSN 2405-884X. Maslin B. (2018). WWW: World Wide Wattle (version 2, Jan 2018). In: Species 2000 & ITIS Catalogue of Life, 2018 Annual Checklist (Roskov Y., Abucay L., Orrell T., Nicolson D., Bailly N., Kirk P.M., Bourgoin T., DeWalt R.E., Decock W., De Wever A., Nieukerken E. van, Zarucchi J., Penev L., eds.). Digital resource at www.catalogueoflife.org/annual-checklist/2018. Species 2000: Naturalis, Leiden, the Netherlands. ISSN 2405-884X Aedo C. (2018). RJB Geranium: Geranium Taxonomic Information System (version Sep 2017). In: Species 2000 & ITIS Catalogue of Life, 2018 Annual Checklist (Roskov Y., Abucay L., Orrell T., Nicolson D., Bailly N., Kirk P.M., Bourgoin T., DeWalt R.E., Decock W., De Wever A., Nieukerken E. van, Zarucchi J., Penev L., eds.). Digital resource at www.catalogueoflife.org/annual-checklist/2018. Species 2000: Naturalis, Leiden, the Netherlands. ISSN 2405-884X.	Use of the content (such as the classification, synonymic species checklist, and scientific names) for publications and databases by individuals and organizations for not-for-profit usage is encouraged, on condition that full and precise credit is given at three levels on all occasions that records are shown.	This online database is copyrighted by Species 2000 on behalf of the Catalogue of Life partners
The Chinese Virtual Herbarium (CVH, http://www.cvh.ac.cn/cvh6/view/index.php)	Biodiversity occurrence data provided by:Chengdu Institute of Biology,Chinese Academy of Sciences;Wuhan Botanical Garden,Chinese Academy of Sciences;Xishuangbanna Tropical Botanical Garden, Chinese Academy of Sciences;Northwest Institute of Plateau Biology,Chinese Academy of Sciences;Guangxi Institute of Botany, Chinese Academy of Sciences;South China Botanical Garden,Chinese Academy of Sciences;Institute of Applied Ecology, Chinese Academy of Sciences;Kunming Institute of Botany, Chinese Academy of Sciences;Lushan Botanical Garden,Chinese Academy of Sciences;Institute of Botany, Jiangsu Province and Chinese Academy of Sciences;Chinese National Herbarium, Institute of Botany, Chinese Academy of Sciences;WUK Herbarium, Northwest Agriculture & Forestry University (Accessed through Chinese Virtual Herbarium(CVH) Data Portal, www.cvh.ac.cn, 2019-04-02)		
The Cichorieae Portal (http://cichorieae.e-taxonomy.net/)	Kilian N., Hand R. & Raab-Straube E. von (eds) 2009 + (continuously updated): *Cichorieae* Systematics Portal. last time accessed 06.12.2019	CC BY 4.0	
The Euro + Med PlantBase (http://ww2.bgbm.org/EuroPlusMed/query.asp)	Euro + Med (2006-): Euro + Med PlantBase - the information resource for Euro-Mediterranean plant diversity. Published on the Internet http://ww2.bgbm.org/EuroPlusMed/. last time accessed 05.06.2019		
The Flora Do Brasil 2020 (http://floradobrasil.jbrj.gov.br/reflora/listaBrasil/ConsultaPublicaUC/ConsultaPublicaUC.do#CondicaoTaxonCP)	Flora do Brasil 2020 under construction. Jardim Botânico do Rio de Janeiro. Available at: < http://floradobrasil.jbrj.gov.br/ > . Accessed on: 03.03.2019	CC BY-SA 4.0	
The Flora Malesiana (https://floramalesiana.org/new/) http://portal.cybertaxonomy.org/flora-malesiana/	The Flora Malesiana, https://floramalesiana.org/new/		
The Integrated Taxonomic Information System (ITIS, https://www.itis.gov/)	Retrieved [October, 10, 2010], from the Integrated Taxonomic Information System on-line database, http://www.itis.gov.	Information presented on the ITIS website is considered public information and may be distributed or copie	
The Melastomataceae database (MEL names, http://www.melastomataceae.net/MELnames/)	Melastomataceae.Net 2007–2020. A Site with Information on the Biodiversity of Melastomataceae: www.melastomataceae.net; assessed: 02.04.2019		
The Pelargonium Page (http://www.pelargonium.si/index.html)	The Pelargonium Page, Matija Strlic, http://www.pelargonium.si/index.html. assessed: 02.04.2019	This work is licensed under a Creative Commons Attribution-NonCommercial-ShareAlike 4.0 International License.	
The Plants Database of the USDA (https://plants.usda.gov/java/)	USDA, NRCS. 2019. The PLANTS Database (http://plants.usda.gov, 23 March 2019). National Plant Data Team, Greensboro, NC 27401-4901 USA		
The Plant List (TPL) (http://www.theplantlist.org)	The Plant List (2013). Version 1.1. Published on the Internet; http://www.theplantlist.org/ (accessed 1st February 2013).	Use of Content for non-commercial or non-profit use is encouraged	
The Smithsonian National Museum of Natural History (https://collections.nmnh.si.edu/search/botany/)	Assessed: 01.08.2019	CC- 0	Information provided with the permission of the National Museum of Natural History, Smithsonian Institution, 10th and Constitution Ave. N.W., Washington, DC 20560-0193. (https://collections.nmnh.si.edu/)
The Southern African plant names and floristic details (SANBI, http://posa.sanbi.org/sanbi)	South African National Biodiversity Institute. 2016. Botanical Database of Southern Africa (BODATSA)	Creative Commons Attribution-NonCommercial-ShareAlike 4.0 International License	
The World Checklist of Selected Plant Families (WCSP, Botanical Gardens Kew, https://wcsp.science.kew.org),	WCSP (2020). ‘World Checklist of Selected Plant Families. Facilitated by the Royal Botanic Gardens, Kew. Published on the Internet; http://wcsp.science.kew.org/ Retrieved	*CC BY*	*Board of Trustees of the Royal Botanic Gardens, Kew*
Grassbase (http://www.kew.org/data/grassbase/index.html)	*Clayton, W.D., Vorontsova, M.S., Harman, K.T. and Williamson, H. (2006 onwards). GrassBase - The Online World Grass Flora*. http://www.kew.org/data/grasses-db.html*. [accessed 08 November 2006]*	CC BY	*Board of Trustees of the Royal Botanic Gardens, Kew*
Palmweb (http://palmweb.org/)	Palmweb [2019]. Palmweb: Palms of the World Online. Published on the internet [http://palmweb.org/]. Acccessed on [05/08/2019].	*CC-BY-NC-SA*	*Board of Trustees of the Royal Botanic Gardens, Kew*
TROPICOS (Missouri Botanical Gardens, http://www.tropicos.org/)	Tropicos.org. Missouri Botanical Garden. 01 Aug 2019 < http://www.tropicos.org >	This site is designed for scientific research by individual researchers. Attribution should be given for use of data derived from the site.	
World Annonaceae (http://annonaceae.myspecies.info)	World Annonaceae, Thomas L.P. Couvreur, http://annonaceae.myspecies.info, assessed: 01.08.2019	CC BY	

**Online-only Table 2:** Freiberg *et al*., LCVP, The Leipzig catalogue of vascular plants, a new taxonomic reference list for all known vascular plants_Description of the options for customization of the fuzzy name matching.

Function argument	Description
genus_search	a logical value, FALSE (default). If TRUE, the function will apply the fuzzy match algorithm also for the search of the genus name, otherwise as default the search is applied only to the epithet, the infraspecies and the name author.
max.distance	an integer value. It represents the maximum distance (number of characters) allowed for a match when comparing the submitted name with the closest name matches in the LCVP.
status	a logical value, TRUE (default). If FALSE, the function will return not only the valid epithet for a species name but also all the possible synonyms.
max.cores	an integer value, indicating the number of CPU cores to be used for the parallelization of the plant name search when a list of plant taxa names is submitted. As default, the maximum number of CPU cores available on the working machine menus one is set.
genus_tab	a logical value, FALSE (default). If TRUE, the function will return the list of plant taxa names belonging to the same genus name submitted by the user.
family_tab	a logical value, FALSE (default). If TRUE, the function will return the list of plant taxa names belonging to the same family name submitted by the user.
order_tab	a logical value, FALSE (default). If TRUE, the function will return the list of plant taxa names belonging to the same order name submitted by the user
infraspecies_tab	a logical value, FALSE (default). If TRUE, the function will return also all the infraspecies names found for a submitted plant name.
encoding	a character vector, “UTF-8” (default). This value will allow the user to set the specific codification of the strings.
out_path	a character vector, which allow the user to define the path where the output file has to be saved. The working directory is set as default.
save	a logical value. FALSE (default). If TRUE, the function will write the output file as comma-separated format (.csv), saving it into the working directory or in the directory already set through the ‘out_path’ option.
visualize	a logical value, TRUE (default). If TRUE the function will visualize the output search on the ‘Source Tab’ of RStudio. This option has to be turned off (FALSE) if the package is executed in a UNIX environment (from the command line) without having a Graphical User Interface.
version	a character vector indicating the current version of the package.

**Online-only Table 3:** Freiberg *et al*., LCVP, The Leipzig catalogue of vascular plants, a new taxonomic reference list for all known vascular plants_Description of the output fields of the name matching function in in the LCVP R Package.

Field name	Description
ID	an integer associated to each taxon; the progression of the numbers follows the same order of the submitted names in the input list (introduced by the user). The only cases where it is possible to find the same ID value is when the searching process for one taxon will return more than one records (e.g. synonyms).
Submitted_Name	a character string of all the input names submitted by the users.
Order	a character string, the corresponding taxonomic order name for the matched taxon name.
Family	a character string, the corresponding taxonomic family name for the matched taxon name.
Genus	a character string, the corresponding taxonomic genus name for the matched taxon name.
Species	a character string, the corresponding taxonomic epithet name for the matched taxon name.
Infrasp	a character string, the corresponding taxonomic infraspecific rank for the matched taxon name.
Infraspecific	a character string, the corresponding taxonomic infraspecific name for the matched taxon name.
Authors	a character string, the description of the authorship for the matched taxon name.
Status	a character string, provides a description if a taxon is classified as ‘valid’, ‘synonym’, or ‘unresolved’.
LCVP_Accepted_Taxon	a character string, provides the accepted (Status = valid) plant taxa name according to the LCVP system.
PL_Comparison	a character string, provides a direct comparison with ‘The Plant List’ (TPL).
PL_Alternative	a character string, provides an alternative name from the TPL.
Score	a character string, a description of the result for the searching process in the LCVP. In this field different information are stored depending by the results of the searching and solving activities (e.g. ‘name found’, ‘genus name not found’, ‘epithet name not found’, misspelling: epithet’, etc.).
Insertion	an integer, describes how many characters the submitted name differs (as inserted types) for the full match with the corresponding name found in the LCVP.
Deletion	an integer, describes how many characters the submitted name differs (as deleted) from the matched name in the LCVP.
Substitution	an integer, describes how many characters the submitted name differs (as substituted) from the matched name in the LCVP.
